# Chimeric Fusion (F) and Attachment (G) Glycoprotein Antigen Delivery by mRNA as a Candidate Nipah Vaccine

**DOI:** 10.3389/fimmu.2021.772864

**Published:** 2021-12-08

**Authors:** Rebecca J. Loomis, Anthony T. DiPiazza, Samantha Falcone, Tracy J. Ruckwardt, Kaitlyn M. Morabito, Olubukola M. Abiona, Lauren A. Chang, Ria T. Caringal, Vladimir Presnyak, Elisabeth Narayanan, Yaroslav Tsybovsky, Deepika Nair, Geoffrey B. Hutchinson, Guillaume B. E. Stewart-Jones, Lisa A. Kueltzo, Sunny Himansu, John R. Mascola, Andrea Carfi, Barney S. Graham

**Affiliations:** ^1^ Viral Pathogenesis Laboratory, Vaccine Research Center, National Institute of Allergy and Infectious Diseases, National Institutes of Health, Bethesda, MD, United States; ^2^ Moderna Inc., Cambridge, MA, United States; ^3^ Vaccine Production Program, Vaccine Research Center, National Institute of Allergy and Infectious Diseases, National Institutes of Health, Bethesda, MD, United States; ^4^ Vaccine Research Center Electron Microscopy Unit, Cancer Research Technology Program, Leidos Biomedical Research, Inc., Frederick National Laboratory for Cancer Research, Frederick, MD, United States; ^5^ Virology Laboratory, Vaccine Research Center, National Institute of Allergy and Infectious Diseases, National Institutes of Health, Bethesda, MD, United States

**Keywords:** Nipah virus (NiV), mRNA, vaccine, Pre-F/G, structure-based immunogen design, pandemic preparedness and response, T cell responses

## Abstract

Nipah virus (NiV) represents a significant pandemic threat with zoonotic transmission from bats-to-humans with almost annual regional outbreaks characterized by documented human-to-human transmission and high fatality rates. Currently, no vaccine against NiV has been approved. Structure-based design and protein engineering principles were applied to stabilize the fusion (F) protein in its prefusion trimeric conformation (pre-F) to improve expression and increase immunogenicity. We covalently linked the stabilized pre-F through trimerization domains at the C-terminus to three attachment protein (G) monomers, forming a chimeric design. These studies detailed here focus on mRNA delivery of NiV immunogens in mice, assessment of mRNA immunogen-specific design elements and their effects on humoral and cellular immunogenicity. The pre-F/G chimera elicited a strong neutralizing antibody response and a superior NiV-specific Tfh and other effector T cell response compared to G alone across both the mRNA and protein platforms. These findings enabled final candidate selection of pre-F/G Fd for clinical development.

## Highlights

- Pre-F and G elicit potent neutralizing antibody responses as mRNA vaccines
- F is the immunodominant antigen eliciting (H2*^d/b^*)-restricted T cell responses
- Coupling precise immunogen design with the mRNA vaccine platform enabled final selection of pre-F/G Fd chimeric design for clinical development

## Introduction

Nipah virus (NiV) is an enveloped non-segmented negative-strand RNA virus in the Henipavirus genus of the *Paramyxoviridae* family (1). Since its emergence in Malaysia in 1998 (1–3), near-annual outbreaks of NiV have occurred in Bangladesh and India (4–10). NiV outbreaks begin with zoonotic exposure to the natural reservoir, fruit bats of the *Pteropodidae* family, or infected intermediate hosts (11–13) as NiV has broad species tropism and can cause disease in a wide range of domestic animals (1, 6, 14–20). NiV infection results in primarily respiratory symptoms with potential neurological manifestations, documented human-to-human transmission and a high mortality rate (60-70%) in recent outbreaks (3, 5, 8, 21–29).

NiV is listed as a high priority pathogen by the World Health Organization (WHO), Centers for Disease Control and Prevention (CDC) and the Coalition of Epidemic Preparedness Innovations (CEPI) (30), and there is a need for medical countermeasures, particularly vaccines. As part of pandemic preparedness efforts, we selected NiV as a prototype paramyxovirus pathogen to optimize antigen design, dissect the humoral and cellular immune responses to vaccination and identify mechanisms of protection. An effective and rapid vaccine response strategy for outbreaks or pandemics requires both precise antigen design and a method for rapid manufacturing and deployment.

Members of the *Paramyxoviridae* and *Pneumoviridae* virus family have two membrane-anchored glycoproteins that are targets for neutralizing antibodies (31), the attachment protein (G, H or HN) and the fusion (F) protein (32). Nipah’s attachment protein is a type II membrane protein that facilitates binding of the NiV virions to the host cells through the ephrin B2/B3 receptors (33–38). The fusion (F) protein utilizes a class I fusion glycoprotein, transitioning from a metastable prefusion conformation (pre-F) to a stable postfusion conformation (post-F) to fuse viral and cellular membranes (39–43) and initiate viral entry, as demonstrated for other class I fusion glycoproteins such as parainfluenza virus (PIV) (44–46) and respiratory syncytial virus (RSV) (47, 48). Based on experience with related paramyxoviruses and pneumoviruses, both the NiV F and G proteins are considered relevant protective antigens and targets for vaccine-elicited neutralizing antibodies. Several recently isolated and characterized monoclonal antibodies have shown F-binding neutralizing antibodies to be pre-F specific (49–52) and five major antigenic sites have been identified on HeV G that inhibit virus by multiple mechanisms and are cross-reactive with NiV G (53, 54). The neutralizing humoral response primarily targets G, which has been the primary focus of vaccine development. A soluble Hendra G recombinant subunit candidate vaccine for Nipah is currently in Phase I clinical evaluation (ClinicalTrials.gov NCT04199169).

Previously, we demonstrated that structure-based antigen design could be used to develop a highly-immunogenic NiV subunit protein vaccine (55). Structure-based design and protein engineering of RSV (47, 48) and PIV (44), specifically to stabilize the fusion protein in its prefusion conformation thereby eliciting a more potent neutralizing antibody response than the post-F conformation and more broadly, stabilization of betacoronavirus spike proteins with the S-2P mutation (56–58) have informed our approach with NiV. Considering prior reports that the henipavirus attachment protein, G, is an important target for humoral response during natural infection, our goal was to design a chimeric vaccine antigen that included both F and G. We focused on stabilizing the fusion protein in its prefusion conformation, designing multimeric forms of G and combining pre-F and G antigens to produce a covalently linked polyprotein. Stabilized pre-F trimer and hexameric G (Hex G) immunogens both induced serum neutralizing activity in mice, while the post-F trimer immunogen did not elicit detectable neutralizing activity. The pre-F trimer covalently linked to three G monomers (pre-F/G) induced responses to both major NiV surface glycoproteins and potent neutralizing activity, making it the lead candidate for clinical development.

Here, we focused on how to rapidly deliver optimized henipavirus immunogens when a pandemic threat arises. Establishing cell lines to express a selected protein and developing purification protocols for clinical-grade subunit protein often takes years, whereas manufacturing nucleic acid vaccines as a platform technology can be achieved in a matter of weeks, as demonstrated with SARS-CoV-2 vaccine, mRNA-1273, an mRNA vaccine developed by Moderna in partnership with NIAID (56, 59). mRNA vaccines have an advantage in manufacturing speed and versatility, potently elicit both humoral and cellular immunity (60–62), have a favorable safety and tolerability profiles (63) and are efficacious (64–67). Two mRNA-based vaccines, mRNA-1273 (Moderna/NIAID) and BNT162b2 (Pfizer/BioNTech), received emergency use authorization at record pace and have been administered to hundreds of millions of people globally. The objectives for these studies include evaluation of mRNA encoding NiV immunogens formulated in lipid nanoparticles (mRNA-LNP), optimization of mRNA immunogen-specific design elements and refinement of the selected candidate for clinical development. We demonstrate how structure-guided antigen design coupled with the mRNA vaccine platform to enable rapid manufacturing forms a strategy to expedite medical countermeasures for future pandemic threats.

## Materials and Methods

### Protein Expression and Purification

NiV F, G, or F/G glycoproteins [described in (55)] were expressed by transfection of 293 Freestyle (293F) cells (Thermo Fisher Scientific, MA) with Turbo293 transfection reagent (SPEED BioSystem, MD) according to the manufacturer’s protocol. Transfected cells were incubated in shaker incubators at 120 rpm, 37°C, 9% CO_2_ overnight. The following day, one tenth culture volume of Cell Booster medium (ABI Scientific, VA) was added to each flask and flasks were incubated for an additional four days in the shaker incubators. Five days post-transfection, cell culture supernatants were harvested and proteins were purified from the supernatants using tandem Ni^2+^ (Roche) and Strep-Tactin (IBA) affinity purification. The C-terminal purification tags were removed by thrombin digestion at room temperature overnight. Proteins were further purified by SEC in a Superdex 200 column (GE) in 1x phosphate-buffered saline (PBS). The strategy for how antigen designs were evaluated and selected was previously reported (55). Briefly, we evaluated expression level, how well and efficiently the protein purified and structural integrity by negative-stain EM.

### Research-Grade Pre-Clinical mRNA and LNP Production Process

A sequence-optimized mRNA encoding Nipah proteins was synthesized *in vitro* using an optimized T7 RNA polymerase-mediated transcription reaction with complete replacement of uridine by N1-methyl-pseudouridine (68). All mRNA immunogens were codon-modified using Moderna’s proprietary codon algorithms designed to improve protein expression and mRNA manufacturability. The reaction included a DNA template containing the immunogen open reading frame flanked by 5′ untranslated region (UTR) and 3′ UTR sequences and was terminated by an encoded polyA tail. After transcription, the Cap 1 structure was added to the 5′ end using vaccinia capping enzyme (New England Biolabs) and Vaccinia 2′ *O*-methyltransferase (New England Biolabs). The mRNA was purified by oligo-dT affinity purification, buffer exchanged by tangential flow filtration into sodium acetate, pH 5.0, sterile filtered, and kept frozen at –20°C until further use.

The mRNA was encapsulated in a lipid nanoparticle through a modified ethanol-drop nanoprecipitation process as described previously (60). In brief, ionizable, structural, helper and polyethylene glycol lipids were mixed with mRNA in acetate buffer, pH 5.0, at a ratio of 2.5:1 (lipids:mRNA). The mixture was neutralized with Tris-Cl pH 7.5, sucrose was added as a cryoprotectant, and the final solution was sterile filtered. Vials were filled with formulated LNP and stored frozen at –70°C until further use. The drug product underwent analytical characterization, which included the determination of particle size and polydispersity, encapsulation, mRNA purity, double stranded RNA content, osmolality, pH, endotoxin and bioburden, and the material was deemed acceptable for *in vivo* study.

### Negative-Stain Electron Microscopy

Proteins were diluted to approximately 0.02 mg/mL with buffer containing 10 mM HEPES, pH 7.0 and 150 mM NaCl, adsorbed to a freshly glow-discharged carbon-coated copper grid, washed with the same buffer, and stained with 0.7% uranyl formate. Datasets were collected using SerialEM (69) on an FEI Tecnai T20 microscope equipped with a 2k x 2k Eagle CCD camera and operated at 200 kV. The nominal magnification was 100,000, corresponding to a pixel size was 0.22 nm. Particles were selected from micrographs automatically using in-house written software (YT, unpublished), followed by manual correction using EMAN2 (70), when necessary. Reference-free 2D classifications were performed with Relion 1.4 (71).

### Thermal Unfolding Transition by Dynamic Light Scattering (DLS)

Samples were diluted in PBS to concentration of 1 mg/mL and filtered with a 0.1 µm, 10 mm diameter PES syringe filter prior to evaluation by Dynamic Light Scattering (DLS) when subjected to a thermal ramp using the DynaPro Plate Reader II (Wyatt Technology, Santa Barbara, CA). Samples were assayed (n = 3) in a 384 well plate; each sample well was filled with 30 µL sample and topped with 10 µL high-purity paraffin oil (Sigma-Aldrich, St. Louis, MO) to prevent evaporation. The wells surrounding the samples were filled with paraffin oil to mitigate edge effects. Each datapoint was generated from 5 readings (5 s acquisition time) for each well during a continuous thermal ramp from 25°C to 80°C @ 0.12°C min^-1^. Particle data were reported for cumulant R_h_ values in the range of 2 – 5000 nm. The thermal transition onset (*T_onset_*) for each sample was determined using the onset function in Dynamics Software, version 7.8.0 (Wyatt Technology, Santa Barbara, CA). Data was not viscosity corrected.

### Differential Scanning Calorimetry (DSC)

Samples were diluted in PBS to concentration of 0.5 mg/mL and Differential Scanning Calorimetry (DSC) thermograms were acquired at 0.5 mg/mL sample concentration using a MicroCal VP-Capillary DSC (Malvern Panalytical, Westborough, PA). Heat differential was monitored as the sample cell temperature was increased from 5°C to 100°C (110°C for the postfusion F protein) at a rate of 60°C/h. Thermograms were subjected to mathematical deconvolution using the MicroCal LLC DSC plug-in for Origin Software (ver. 7.0) to resolve underlying peaks and determine transition midpoints (59). Buffer-subtraction and baseline correction were applied.

### Animal Immunizations

All animal experiments were reviewed and approved by the Animal Care and Use Committee of the Vaccine Research Center, NIAID, NIH and all animals were housed and cared for in accordance with local, state, federal and institute policies in an American Association for Accreditation of Laboratory Animal Care (AAALAC)-accredited facility at the NIH. Groups of 10 CB6F1/J female mice (Jackson Laboratory) were immunized twice at weeks 0 and 3 intramuscularly with 10 μg of recombinant NiV post-F, pre-F, G or pre-F/G chimeric designs combined with 100 μg aluminum hydroxide (alum) in a volume of 100 μL (50 μL/leg) or 0.1 μg, 1 μg, 3 μg or 10 μg mRNA in a volume of 50 μL in the right leg. Serum was collected at weeks 2/3, 5/6 and/or 9 following immunization (no more than 100 μL/tail bleed). Week 5/6 or 9 sera was assessed for immunogenicity in enzyme-linked immunosorbent assays and for neutralization in VSVΔG-luciferase pseudovirus neutralization assay *in vitro*.

### Endpoint ELISA to Measure Pre-F and Monomeric G-Specific Responses

Immulon 4HBX 384-well ELISA plates (Thermo Scientific) were coated with 40 ng/well of NiV Pre-F or NiV monomeric G protein in BupH buffer (Pierce) at 4°C for 16h. The NiV pre-F protein used to coat ELISA plates is the same antigen used in immunization studies (also contains thrombin-his-strep tag) while the NiV monomeric G protein has the thrombin-his-strep tag and no multimerization domain, differing from the antigen used in immunization studies. After standard washes and blocks, plates were incubated with 4xfold serial dilutions of heat-inactivated sera for 30-45 min at room temperature. Following washes, anti-mouse IgG-horseradish peroxidase conjugates (Sigma) were used as secondary antibody and 3,5,3’,5’-tetramethylbenzidine (TMB) (KPL) was used as the substrate to detect antibody responses. Endpoint titers were calculated as the dilution that emitted an optical density exceeding background (secondary antibody alone) with a predetermined absorbance cut-off of 0.2 (approximately 3-4x average background value).

### Generation of NiV Pseudovirus

To obtain VSVΔG-luciferase pseudotyped with NiV F_WT_ and NiV G_WT_ proteins, BHK21 cells were first co-transfected with VRC8400 NiV F_WT_ and VRC8400 NIV G. Transfected cells showing extensive cell-to-cell fusion were infected with VSV-G complemented with VSVΔG-luciferase at an MOI of 4, about 24 hours post-transfection. At 1 hour post-infection, input virus was removed, cells were washed with 1xPBS and DMEM with 10% FBS, 1% Pen/Strep, 1% GlutaMax was added to the cells. Medium/cells containing VSVΔG-luciferase pseudotyped with NiV F_WT_ and G was collected after 24 hours and sonicated, before being clarified. Stock pseudovirus was confirmed to have incorporated both NiV F and NiV G by demonstrating h5B3 mAb and m102.4 mAb were able to neutralize pseudovirus infectivity individually in a luciferase assay.

### Imunogenicity of NiV F, G, and F/G Chimeric Designs in Mice

A pseudovirus neutralization assay is used because NiV is classified as a BSL-4 pathogen. Neutralizing antibody titers were determined using a microneutralization assay using VSVΔG-luciferase expressing NiV F and NiV G in Vero E6 cells as previously described (72). NiV F/G VSVΔG-luciferase pseudovirus was first incubated with anti-VSV G 8G5 monoclonal antibody (Kerafast) for 15 min to neutralize any trace infection due to residual VSV G that may have been incorporated into the particles pseudotyped with NiV F and G proteins. Serum samples were heat-inactivated at 55°C for 30 min. Serum samples or pooled serum samples from each immunization group were serially diluted in DMEM with 10% FBS, 1% Pen/Strep, 1% GlutaMax and mixed with equal volume of pseudotyped particles with anti-VSV G 8G5 monoclonal antibody, incubated for 30 min at room temperature before addition to Vero E6 cells. After 24 hours, medium was removed by aspiration, plates were washed with 300 μL 1xPBS/well. Cell lysis and detection of firefly luciferase were performed according to the protocol recommended by the manufacturer (Promega Inc). Briefly, firefly luciferase assay lysis buffer was thawed to room temperature, diluted 1:5 with ddH_2_O and 20 μL was added to each well. Plates were placed on an orbital shaker for 20-30 min. Following lysis, 50 μL of luciferase assay reagent was added to each well and read at 570 nm on the SpectraMax L luminometer (Molecular Devices). Percent neutralization was normalized considering uninfected cells as 100% neutralization and cells infected with only pseuodvirus as 0% neutralization. The 80% inhibitory concentration (IC_80_) was calculated by curve fitting and non-linear regression (log(agonist) *vs* normalized response (variable slope) ECAnything) in triplicate wells using GraphPad Prism v8.

### T Cell Peptide Libraries

Peptides spanning the ectodomain of F and G (15-mers overlapping by 11 amino acids) were synthesized by JPT (F and G peptide pools, 85% pure). The F pool has 123 peptides and the G pool has 108 peptides.

### Activation-Induced Marker Assay (AIM)

Mononuclear single cell suspensions from whole mouse spleens were generated using a gentleMACS tissue dissociator (Miltenyi Biotec) followed by 70 µm filtration and density gradient centrifugation using Fico/Lite-LM medium (Atlanta Biologicals). Antigen-specific CD8^+^ and CD4^+^ T cells, including Tfh cells were examined using an activation induced marker (AIM) assay. Splenocytes were resuspended in R10 media containing BD Fc Block and anti CD154/CD40L antibody conjugated to APC (BD, clone: MR1) and incubated for 6 hr at 37°C under three conditions: no peptide (DMSO only) stimulation, and stimulation with the F and G peptide pools. Peptide pools were used at a final concentration of 2 µg/ml each peptide. Cells from each group were pooled for stimulation consisting of a 6 hr incubation with 1x eBiosience cell stimulation cocktail containing PMA and ionomycin (Invitrogen), according to manufacturer’s instructions as a positive control. Following stimulation, cells were washed with PBS prior to staining with LIVE/DEAD Fixable Blue Dead Cell Stain (Invitrogen) for 20 min at RT. Cells were then washed in FC buffer (PBS supplemented with 2% HI-FBS and 0.05% NaN_3_) and resuspended in BD Fc Block (clone 2.4G2) for 5 min at RT prior to staining with a surface stain cocktail containing the following antibodies purchased from BD and Biolegend: CD3 (17A2) BUV737, CD4 (RM4-5) BV480, CD8 (53-6.7) BUV805, I-A/I-E (M5/114.15.2) PE, CD44 (IM7) BUV395, CD62L (MEL-14) PE-Cy7, CXCR5 (2G8) PE-CF594, PD-1 (J43) BV421, CD69 (H1.2F3) BV605. After 15 min at RT, cells were washed in FC stain buffer solution and resuspended in 0.5% PFA-FC stain buffer prior to running on a Symphony A5 flow cytometer (73). Analysis was performed using FlowJo software, version 10.6.2 according to the gating strategy outlined in **Supplemental Figure 6**. Background cytokine expression in the no peptide condition (DMSO) was subtracted from that measured in the F and G peptide pools for each individual mouse, with representative upregulation of activation markers (CD69 and CD40L) shown in **Supplemental Figure 7**.

## Results

### Evaluation of mRNA Dose Response 

**Figure 1:**
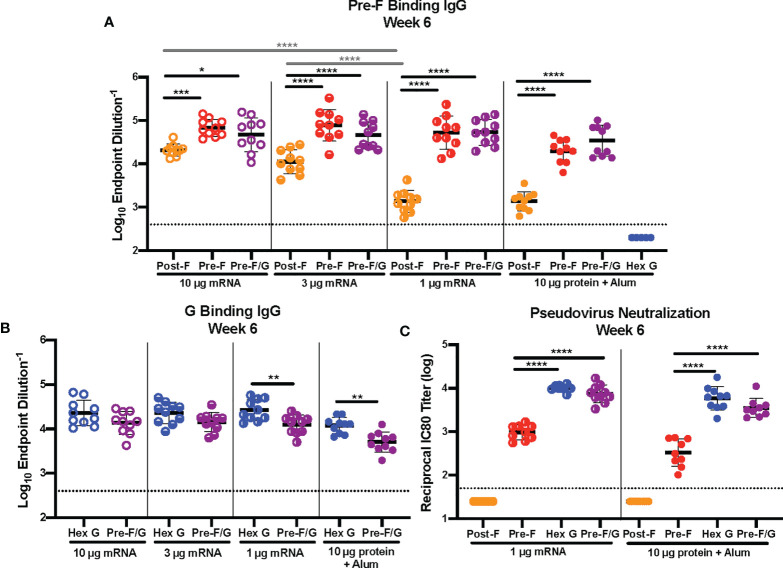
mRNA Dose-Response Study. **(A, B)** Serum samples were assessed for NiV pre-F specific IgG **(A)** or monomeric G-specific IgG **(B)** by enzyme-linked immunosorbent assay (ELISA). Line represents mean of all animals in each group +/- standard deviation. **(C)** VSVΔG-luciferase pseudovirus neutralization assays were performed on individual mouse sera collected at week 6. The log_10_ reciprocal IC_80_ neutralization titers for each sample was calculated by curve fitting and non-linear regression using GraphPad Prism. Line represents mean of log_10_ reciprocal IC_80_ dilution +/- standard deviation. P values were calculated using two-way ANOVA with Tukey’s multiple comparisons test (*p < 0.05, **p < 0.01, ***p < 0.001, ****p < 0.0001). Dotted lines represent assay limits of detection.

We first evaluated the dose response to mRNA-LNP encoding NiV antigens. CB6F1/J mice (H2^*d/b*^) were immunized intramuscularly with either 1 μg, 3 μg or 10 μg mRNA expressing pre-F, post-F, Hex G or pre-F/G or 10 μg protein, adjuvanted with aluminum hydroxide (alum) at weeks 0 and 3 (**Supplemental Figure 1**). Three weeks post-second immunization, sera were assessed for binding to pre-F or monomeric G antigens by enzyme-linked immunosorbent assay (ELISA). Mice immunized with pre-F, post-F or pre-F/G mRNA had robust F-specific antibody responses (**Figure 1A**) regardless of mRNA dose, while mice immunized with Hex G protein did not elicit detectable F-specific antibodies. Consistent with our observations with protein immunogens (55), mRNA designs incorporating pre-F demonstrated superior elicitation of F-specific antibodies compared to those expressing post-F. The F-specific antibody response measured by ELISA in post-F mRNA immunized mice was dose-dependent with a ELISA endpoint geometric mean titer (GMT) of 1:22,000 in the 10 μg mRNA dose group compared to 1:1,600 in the 1 μg mRNA dose group. In contrast, pre-F and pre-F/G mRNA immunized mice had uniformly high F-specific endpoint GMT (greater than 1:65,000 at all mRNA doses).

A similar outcome was noted for G-specific antibody responses in mice immunized with Hex G or pre-F/G mRNA (**Figure 1B**) with ELISA endpoint GMT of 1:28,000 at 10 μg and 1:30,000 at 1 μg mRNA dose groups or 1:16,000 at 10 μg and 1:14,000 at 1 μg mRNA dose groups, respectively. The difference in G-specific antibody levels between Hex G and pre-F/G may be related to the molar ratio of G in each design and G monomer valency; Hex G is composed of six G monomers while pre-F/G has three G monomers.

Next, we measured the ability of NiV mRNA-LNP vaccines to elicit neutralizing antibodies using a NIV F/G VSVΔG-luciferase pseudovirus system, described previously (74). Mouse sera were serially diluted for the 1 μg mRNA and 10 μg alum adjuvanted protein immunized groups (**Figure 1C**). No detectable neutralizing activity was observed in sera from mice immunized with post-F (mRNA or protein) while mice immunized with pre-F, Hex G or pre-F/G (mRNA or protein) all had neutralizing activity. For each mRNA/protein antigen design, the 1 μg mRNA group reciprocal IC_80_ neutralization GMT was 40-60% higher than 10 μg of the corresponding protein. While there is no statistically significant difference in the measured neutralizing activity between Hex G and pre-F/G, they both elicit ten-fold more neutralizing antibodies than pre-F alone (**Figure 1C**). Our results demonstrate that both pre-F and G-specific antibodies contribute to neutralizing activity and confirm that G is the primary target for neutralization.

### mRNA-Immunogen Design Elements Like Signal Peptide and Protein Solubility Does Not Affect Immunogenicity

**Figure 2:**
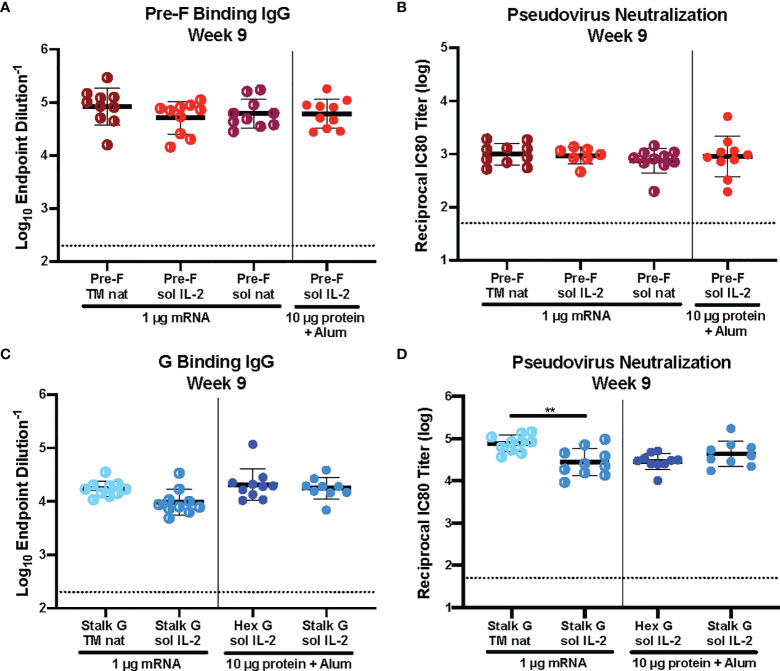
Immunogenicity of mRNA Immunogen-Specific Design Elements. **(A, C)** Serum samples 6 weeks post-boost were assessed for NiV pre-F specific IgG **(A)** or monomeric G-specific IgG **(C)** by ELISA. Line represents mean of all animals in each group +/- standard deviation. **(B, D)** VSVΔG-luciferase pseudovirus neutralization assays were performed on individual mouse sera collected 6 weeks post-boost. The log_10_ reciprocal IC_80_ neutralization titer for each sample was calculated by curve fitting and non-linear regression using GraphPad Prism. Line represents mean of log_10_ reciprocal IC_80_ dilution +/- standard deviation. P values were calculated using one-way ANOVA with Tukey’s multiple comparisons test (**p < 0.01). Dotted lines represent assay limits of detection.

Historically, immunogen design elements like signal peptide and protein solubility have played a role in immunogenicity. We asked whether we could improve immunogenicity by altering the signal peptide (IL-2 *vs* native) or protein solubility (secreted *vs* membrane-anchored) of our pre-F and G mRNA immunogens. CB6F1/J mice (H2^*d/b*^) were immunized intramuscularly with 1 μg mRNA or 10 μg protein, adjuvanted with alum expressing various pre-F or G designs at weeks 0 and 3 (**Supplemental Figure 2**). Six weeks post-second immunization, we assessed binding to pre-F or monomeric G antigens by ELISA and pseudovirus neutralization (**Figure 2**). There was no statistically significant difference in pre-F specific antibodies (**Figure 2A**) or neutralizing antibody titers (**Figure 2B**) elicited by pre-F mRNA designs with different signal peptides (native *vs* IL-2) or with secreted *vs* membrane-anchored designs. Membrane-anchored NiV G mRNA with the native signal peptide (wild type G, Stalk G TM nat) elicited more neutralizing antibodies than secreted NiV G mRNA with the IL-2 signal peptide (Stalk G IL-2 sol) with reciprocal IC_80_ neutralization GMT for Stalk G TM nat of 85,000 and for Stalk G sol IL-2 of 36,000 in the 1 μg mRNA dose groups (**Figure 2D**). Although the difference in G-specific binding antibodies was not statistically significant between the two mRNA designs (**Figure 2C**), binding antibodies elicited with the Stalk G TM nat mRNA trended higher than the Stalk G sol IL-2 mRNA with ELISA endpoint GMT for Stalk G TM nat of 1:68,000 and for Stalk G sol IL-2 of 1:31,000 in the 1 μg mRNA dose group.

**Figure 3:**
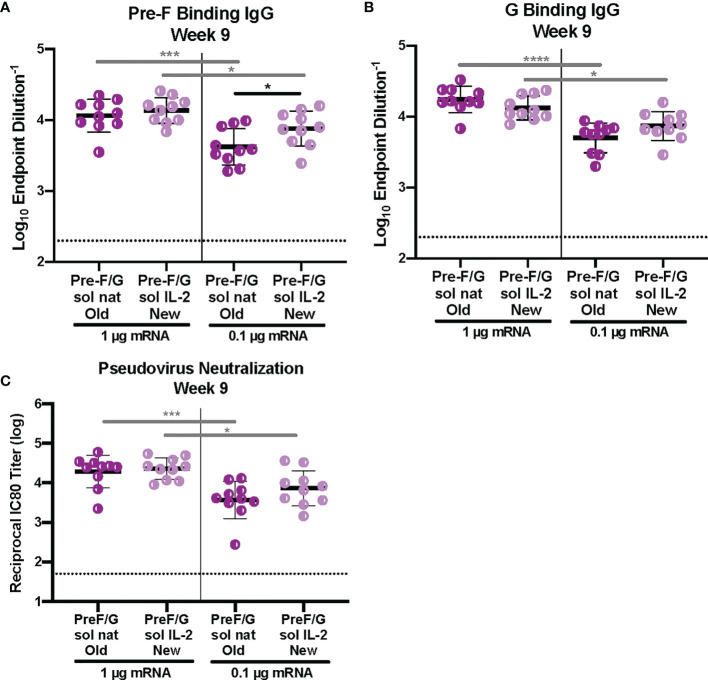
Immunogenicity of mRNA Pre-F/G Chimeric Design Elements. **(A, B)** Serum samples 6 weeks post-boost were assessed for NiV pre-F specific IgG **(A)** or monomeric G-specific IgG **(B)** ELISA. Line represents mean of all animals in each group +/- standard deviation. **(C)** VSVΔG-luciferase pseudovirus neutralization assays were performed on individual mouse sera collected 6 weeks post-boost. The log_10_ reciprocal IC_80_ neutralization titer for each sample was calculated by curve fitting and non-linear regression using GraphPad Prism. Line represents mean of log_10_ reciprocal IC_80_ dilution +/- standard deviation. P values were calculated using two-way ANOVA with Tukey’s multiple comparisons test (*p < 0.5, ***p < 0.001, ****p < 0.0001). Dotted lines represent assay limits of detection. All mRNA immunogens were codon-modified using Moderna’s proprietary codon algorithms designed to improve protein expression and mRNA manufacturability. In this figure, we specifically evaluated mRNA preparations that had used two different codon algorithms, referred to as “old” and “new”.

Next, we examined the role of signal peptide and codon usage on our pre-F/G chimeric design. All mRNA immunogens were codon-modified using Moderna’s proprietary codon algorithms designed to improve protein expression and mRNA manufacturability. CB6F1/J mice (H2^*d/b*^) were immunized intramuscularly with 1 μg or 0.1 μg mRNA or 10 μg protein, adjuvanted with alum expressing various pre-F/G designs at weeks 0 and 3 (**Supplemental Figure 3**). Six weeks post-second immunization, we assessed immunogenicity (**Figure 3**). All mice had robust F-specific (**Figure 3A**) and G-specific (**Figure 3B**) binding and neutralizing antibody responses (**Figure 3C**). Pre-F/G mRNA induced dose-dependent binding and neutralizing antibody response, but no statistically significant difference between the two mRNA designs was observed suggesting that neither the signal peptide nor the codon optimization algorithm affected immunogenicity (**Figures 3A–C**).

### Trimerization Domain Does Not Affect Immunogenicity of Pre-F/G Chimera mRNA

We set out to further refine the pre-F/G chimeric design for clinical development. The initial pre-F/G immunogen included both GCN4 and T4-phage fibritin (Fd) trimerization domains. Previously, DS-Cav1 protein, an RSV stabilized pre-F trimerized using the Fd trimerization domain, was evaluated in a Phase I clinical trial (VRC 317) (75, 76). Therefore, we aimed to evaluate immunogens that incorporated only GCN4 or Fd for stability and immunogenicity.

**Figure 4:**
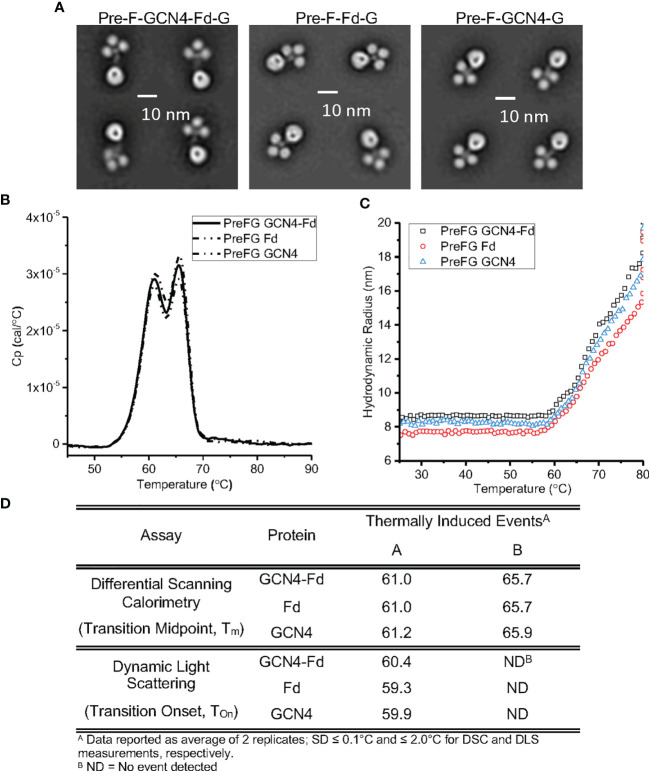
Biophysical Properties of Pre-F/G Chimeric Proteins with Different Trimerization Domains. **(A)** Negative-stain EM analysis of NiV pre-F/G chimeric proteins. **(B, C)** Thermodynamic and colloidal stability assessment of pre-F/G chimeric trimerization domains assessed by **(B)** differential scanning calorimetry (DSC) and **(C)** dynamic light scattering (DLS). **(D)** Summary of DSC and DLS data.

We assessed the conformational and colloidal stability of the pre-F/G GCN4-Fd protein compared to pre-F/G GCN4 and pre-F/G Fd proteins. Analysis by negative-stain electron microscopy showed similar protein conformation and architecture composed of clearly defined pre-F and G globular head components (**Figure 4A**). Differential scanning calorimetry thermograms (**Figures 4B, D**) were similar for all three variants. Two transition midpoints were observed at approximately 61°C and 66°C, corresponding to the primary transition midpoints of pre-F and G proteins, respectively, suggesting minimal interaction between the two domains within the chimera and thermal stability of the covalent interaction, as previously described (55). Colloidal stability of the three proteins, as assessed by dynamic light scattering analysis (**Figures 4C, D**), was also similar, with transition onset (T_onset_) values of ~ 59°C-61°C) suggesting that the first DSC T_m_ coincides with a heat-induced aggregation event. The biophysical characterization of the three pre-F/G chimeric proteins with different trimerization domains indicate identical conformational and colloidal stability properties.

**Figure 5:**
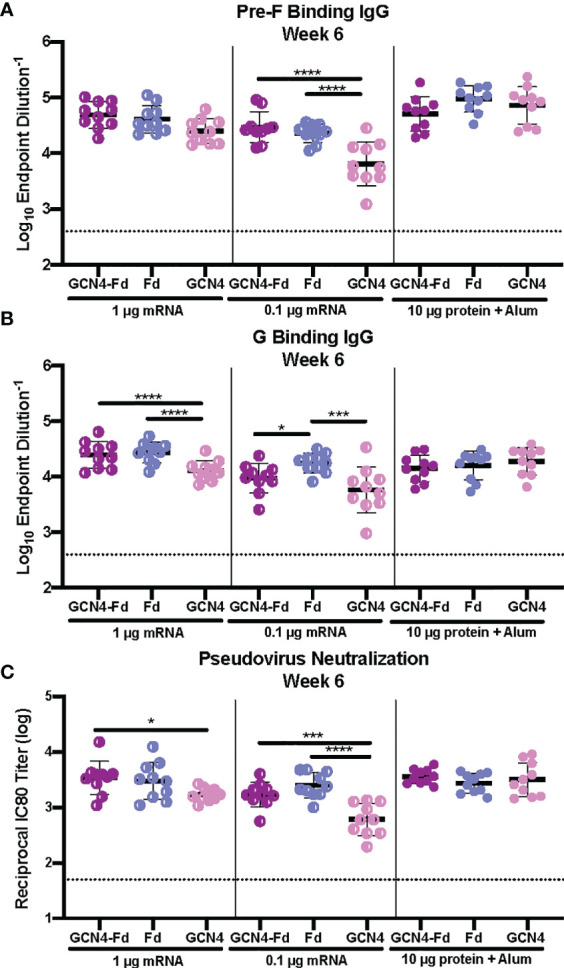
Immunogenicity of Pre-F/G Chimeric mRNA Trimerization Domain Designs. **(A, B)** Serum samples 3 weeks post-boost (week 6) were assessed for NiV pre-F specific IgG **(A)** or monomeric G-specific IgG **(B)** by ELISA. Line represents mean of all mice/group +/- standard deviation. **(C)** VSVΔG-luciferase pseudovirus neutralization assays were performed on individual mouse sera collected at week 6. The log_10_ reciprocal IC_80_ titer for each sample was calculated by curve fitting and non-linear regression using GraphPad Prism. Line represents mean of log_10_ reciprocal IC_80_ dilution +/- standard deviation. P values were calculated using two-way ANOVA with Tukey’s multiple comparisons test (*p < 0.05, ***p < 0.001, ****p < 0.0001). Dotted lines represent assay limits of detection.

Next, we examined the role of the trimerization domain in pre-F/G on immunogenicity. CB6F1/J mice (H2^*d/b*^) were immunized with either 1 μg or 0.1 μg mRNA or 10 μg protein, adjuvanted with alum at weeks 0 and 3 (**Supplemental Figure 4**). At week 6, binding to pre-F and monomeric G antigens (**Figures 5A, B**) as well as neutralization (**Figure 5C**) were assessed. All animals had robust F- and G-specific antibody responses and elicited neutralizing antibodies. There was no difference in the antibody binding or neutralizing antibody titers between the pre-F/G GCN4-Fd and pre-F/G Fd chimeric immunization groups (**Figures 5A–C**). However, mRNA encoding the pre-F/G chimera using GCN4 only elicited three-fold lower antibody responses than pre-F/G GCN4-Fd or pre-F/G-Fd at the 0.1 μg mRNA dose **Figures 5A–C**). We found no difference in the biophysical properties or immunogenicity between the original pre-F/G GCN4-Fd chimera and pre-F/G Fd design.

### Pre-F/G Chimera Demonstrates Superior Elicitation of NiV-Specific T Cell Responses Compared to G

We hypothesized that the pre-F/G chimera would elicit broader and more diverse T cell responses than either pre-F or Hex G alone, suggesting an advantage to including F in the lead candidate design. We analyzed antigen-specific T cell responses elicited by pre-F/G vaccination compared to pre-F or Hex G alone, employing an activation-induced marker (AIM) assay in conjunction with peptide pool restimulation over a 6 hour culture period. The AIM assay detects antigen-specific T cells from the endogenous, polyclonal repertoire that upregulate the activation markers CD40L(CD154) and CD69 in response to peptide stimulation (63, 77). Peptide libraries (15-mers overlapping by 11 amino acids) spanning the entire ectodomain of the F or G coding regions were used to quantify the F and G-specific responses, not to map specific epitopes. The AIM assay gating strategy, antibody panel, and representative data are shown in **Supplemental Figures 6**, **7**.

CB6F1/J mice (H2^*d/b*^) were immunized intramuscularly with either 1 μg mRNA or 10 μg protein, adjuvanted with alum expressing pre-F, Hex G or pre-F/G GCN4-Fd at weeks 0 and 3 (**Supplemental Figures 5A, B**). Week 6 sera was assessed for binding to pre-F or monomeric G antigens and neutralizing antibody activity. Hex G and pre-F/G mRNA immunogens elicited equivalent neutralizing antibody responses at 1 μg mRNA dose (**Supplemental Figures 5C–E**), similar to previous studies. Four weeks post-boost, spleens from 5 mice/group were harvested for T cell analyses. The pre-F/G chimera vaccine elicited CD4^+^ T cell responses to F and G independent of vaccine delivery platform (**Supplemental Figure 8A**). CD4^+^ T follicular helper cells (Tfh) specific to F and G were also induced (**Supplemental Figure 8B**). CD8^+^ T cell responses were also detected only in mRNA immunized groups (**Supplemental Figure 8C**). F-specific T cell responses were dominant compared to G-specific responses irrespective of T cell subset (CD4^+^ *vs* CD8^+^) when delivered by mRNA (**Supplemental Figures 8A-C**). Immunization with 1 µg pre-F/G mRNA resulted in significantly more F-specific CD4^+^ T cells, including Tfh (**Supplemental Figures 8A, B**) and increased CD8^+^ T cell responses (**Supplemental Figure 8C**) compared to immunization with Hex G.

**Figure 6:**
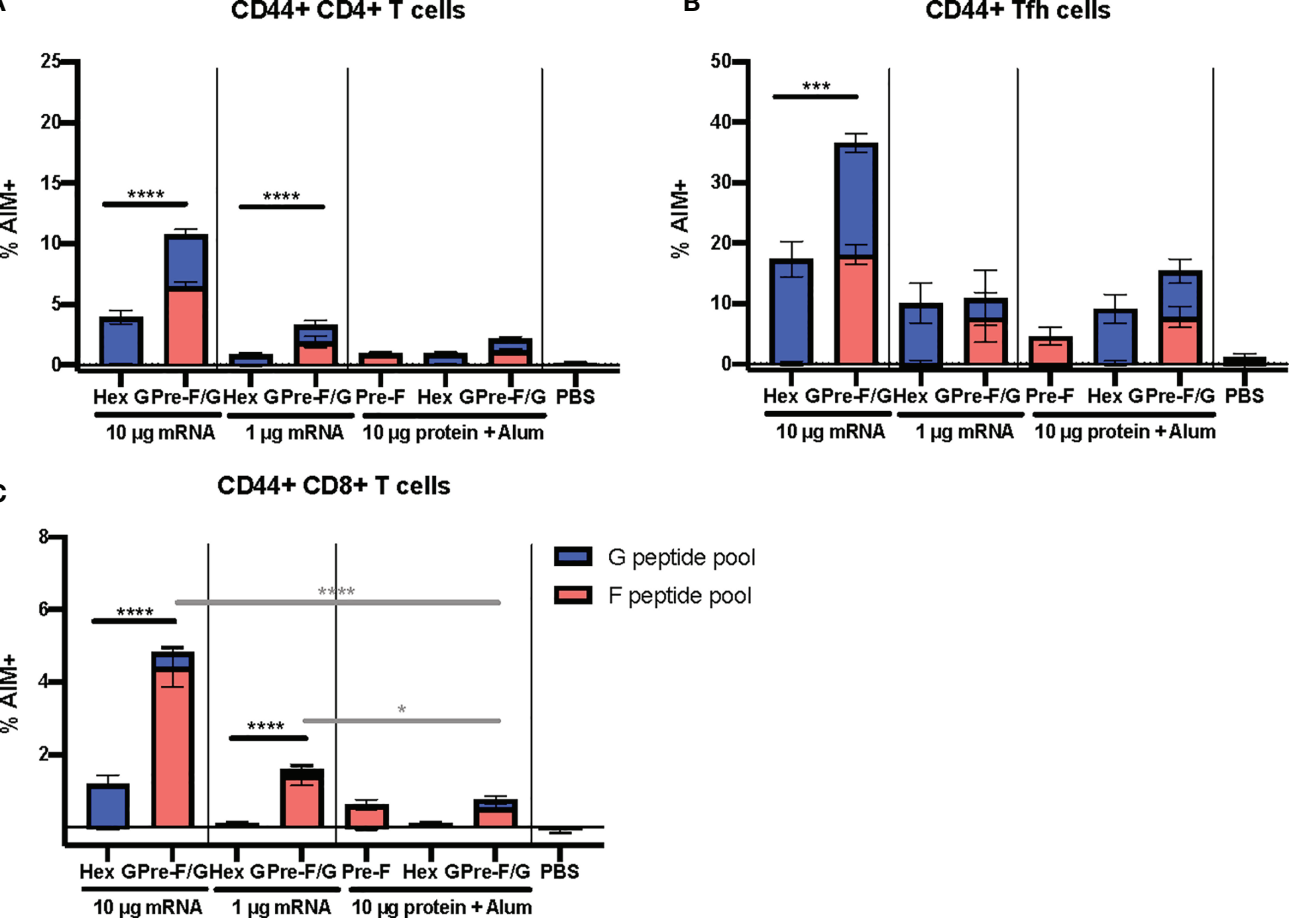
Cellular Immune Responses to F and G. **(A)** Antigen-experienced (CD44^+^) CD4^+^ **(B)** Tfh (CXCR5^+^ PD-1^+^), and **(C)** CD8^+^ T cell responses determined by AIM assay (CD40L and CD69 upregulation). N = x/gr and error bars reflect SEM. 10 mice/group were analyzed. P values were calculated using two-way ANOVA with Tukey’s multiple comparisons test (*p < 0.05, ***p < 0.001, ****p < 0.0001), comparing F peptide pool only % AIM^+^ cells.

The pre-F/G chimera demonstrates a superior elicitation of NiV-specific Tfh and other effector T cells compared to G alone across both the mRNA and protein platforms. To further evaluate the T cell responses to pre-F/G chimera compared to Hex G, CB6F1/J mice (H2^*d/b*^) were immunized intramuscularly with either 1 μg or 10 μg mRNA or 10 μg protein, adjuvanted with alum expressing Hex G or pre-F/G Fd at weeks 0 and 3 (**Supplemental Figures 9A, B**). Week 5 sera was assessed for binding to pre-F or monomeric G antigens and neutralizing antibodies. Again, Hex G and pre-F/G mRNA immunogens elicited equivalent neutralizing antibody responses (**Supplemental Figures 9C–E**). Two weeks post-boost, spleens were harvested to evaluate T cell responses. The pre-F/G chimera elicited CD4^+^ T cell responses, CD8^+^ T cells and induced CD4^+^ Tfh cells to both F and G in a mRNA dose-dependent manner (**Figures 6A–C**). Immunization with either dose of pre-F/G mRNA resulted in significantly more CD8^+^ T cells than the 10 µg of the same protein (**Figure 6C**), in agreement with other reports demonstrating stronger CD8^+^ T cell induction *via* nucleic acid immunization than subunit protein (78, 79). The increased breadth and frequency of T cell responses elicited by pre-F/G immunogens combined with neutralizing antibody response to both glycoproteins indicate an advantage of including both in the selected immunogen.

## Discussion

Coupling structure-based precision antigen design with the rapid, adaptable and scalable mRNA vaccine platform provides a powerful tool to respond quickly and effectively to pandemic threats. The value of this approach is increased if antigen design strategies can be applied to other strains within a virus family or genus as previously demonstrated for coronaviruses (56) and henipaviruses (55). The studies presented here demonstrate that mRNA is a robust and viable vaccine platform for NiV immunogens. While Hex G and pre-F/G both elicit potent humoral responses, inclusion of pre-F in the pre-F/G chimeric design increases the breadth of antibody and T cells responses (CD4^+^, Tfh, CD8^+^), decreasing the potential for immune escape. Elements of both the protein and mRNA were refined to enable selection of the pre-F/G Fd candidate mRNA vaccine for clinical development.

Our findings with mRNA antigen delivery were consistent with previous findings with protein delivery; NiV pre-F induced more potent neutralizing activity than post-F and Hex G elicited similar neutralizing activity as the pre-F/G chimera (55). We did not see a dose-response in binding antibodies or neutralizing activity for mRNA doses between 10 μg and 1 μg for pre-F, Hex G or pre-F/G immunogens, however, as the dose was dropped to 0.1 μg in subsequent experiments, the immunogenicity was diminished suggesting that the dose threshold for inducing a maximum antibody response in mice is <1 μg. Mice immunized with post-F mRNA, which is inherently less immunogenic, showed a dose-response from 3 μg to 1 μg in F-specific binding antibody. Optimization studies for pre-F and G design elements were tested at 1 μg mRNA dose and while evaluating those designs at levels above the dose-threshold may not allow for detection of small immunogenic differences, detection of substantial enhancing or detrimental differences would be possible. Other mRNA studies in mice have shown a dose-response for IC_50_ neutralization titers for mRNA-1273 (56) and in HAI titer for A/Cal09 HA mRNA-LNP (80) in the 1 μg range whereas mRNA encoding ZIKV IgE_sig_-prM-E (81) or MERS-CoV spike (56) based on EC_50_ neutralizing activity did not show a significant decrement in immunogenicity until the 0.1 μg dose range.

Several aspects of mRNA design features can affect vaccine immunogenicity. Here, we explored modifications of the signal peptide and transmembrane domain. Unlike prior studies demonstrating that the IL-2 signal peptide could improve protein expression when the native signal peptide efficiency was low or unknown (82), use of the IL-2 signal peptide did not confer an advantage over the native signal peptide for the pre-F, G or pre-F/G mRNA. Similarly, retention of the transmembrane domain has been shown to improve immunogenicity for other class I fusion proteins, such as the spike protein of MERS-CoV (56), but did not improve immunogenicity of NiV F and marginally, improved the immunogenicity of the NiV G. Protein stability and retention of the prefusion conformation and maintenance of the neutralization-sensitive epitopes may be an important determinant of secreted protein immunogenicity. The NiV pre-F structural integrity was maintained over a large thermal range (55) and may explain the equivalent immunogenicity of secreted and membrane-anchored versions. Our findings support empirically testing each antigen for optimal design characteristics and immunogenicity profiles.

These studies focused on vaccine immunogenicity, and subsequent studies will evaluate efficacy. Several well-characterized animal models exist for testing vaccine efficacy against NiV, including Syrian golden hamsters, ferrets and African green monkeys which most accurately recapitulate the neurological and respiratory pathology seen with NiV infection in humans (18, 83–85). Efficacy studies are challenging because NiV is classified as a BSL-4 pathogen and a limited number of facilities have capabilities to perform such studies. As mentioned previously, mRNA delivery of vaccine antigens has been well tolerated in humans (64–66). The NiV pre-F antigen has not been tested in humans but extensive testing of a prefusion stabilized RSV F protein containing the foldon trimerization domain (DS-Cav1) has been evaluated in a Phase I clinical trial with no toxicity reported from the protein antigen (75, 76). A Hendra soluble G protein is currently under evaluation in a Phase I clinical trial, but data on toxicity has not yet been reported. Additionally, Soltan et al. analyzed the whole Nipah proteome using immunoinformatic and computational prediction tools to design a multitope vaccine that predicted neither NiV F nor G proteins were toxic or allergenic (86).

A complicated but critical aspect of vaccine development is delineating correlates of protection. Currently, no clear correlates of protection have been defined in either animal models or humans for Nipah or other henipaviruses. Humoral responses are clearly important in animal models such as hamster, ferret and non-human primates where passive transfer of immune serum or administration of virus-specific monoclonal antibodies provides protection from NiV challenge (87–90) but few animal studies have assessed T cell responses. In two human survivors from the 2018 Kerala outbreak, longitudinal analysis of cell-mediated and humoral immune responses to NiV infection during the acute and convalescent phases showed absolute B- and T-cell counts remained largely within normal limits. However, significant activation of CD8^+^ T lymphocytes was observed, coinciding with viral clearance (91) suggesting a role for CD8^+^ T cells. While our studies have shown that both Hex G and pre-F/G chimera elicit comparable levels of neutralizing activity, the inclusion of F increases the number of neutralizing epitopes and in H2*^d/b^* mice substantially increases the elicitation of both CD4^+^ and CD8^+^ T cell responses. There are multiple mechanisms reported by which NiV infection antagonizes innate immune responses, but to date, none have been attributed to F or G. Henipaviruses encode several proteins that block innate immune responses and that these proteins likely serve as virulence factors (92–94). NiV M, P, V, W and C proteins have all demonstrated the ability to interfere with type I interferon induction and effector functions (94–98).

Selection of the pre-F/G Fd chimeric mRNA design as the lead candidate for clinical development is based on increased antigenic breadth for both neutralizing antibodies and Tfh when both F and G antigens are included and the induction of both robust neutralizing activity and CD8^+^ T cell responses when delivered by mRNA. Additionally, inclusion of F as well as G in the lead candidate is also supported by work from Stroh et al. They showed VLPs composed of the NiV surface glycoproteins G and F and the matrix protein of the closely related Hendra virus (HeV M) included to promote the formation of the VLP, induced both neutralizing antibodies and antigen-specific CD8^+^ T cell responses in C57BL/6 mice. The combination of all three proteins (NiV G, NiV F and HeV M) was important for increasing CD8^+^ T cell responses in C57BL/6 mice, suggesting that VLPs with greater antigenic content may provide better immunity (99).

We have focused on Nipah virus as a prototype for paramyxoviruses. This is part of a larger pandemic preparedness strategy involving the development of generalizable vaccine antigen design solutions for prototype pathogens from each virus family known to infect humans (100). We used our previous knowledge of protein engineering approaches for stabilizing class I fusion proteins of RSV and PIV and applied them to NiV F protein. As the primary target for neutralizing antibodies against members of the *Paramyxoviridae* family may be either the fusion protein (F) or the attachment protein (G, H, or HN), an immunogen that includes both antigens is important for a generalizable design that could be applied to other family members as needed. For NiV, the primary target for neutralizing antibody is G, but as shown here, the addition of F stabilized in its prefusion conformation provides several potential advantages over a vaccine with G only.

A pandemic response requires both a precision-designed antigen and a delivery platform that is safe and can be rapidly manufactured at large-scale such as mRNA. Several elements of the National Institute of Allergy and Infectious Diseases (NIAID) Prototype Pathogen Approach for Pandemic Preparedness and Response Program were applied to Nipah vaccine development combining structure-based immunogen design at the Vaccine Research Center and mRNA vaccine platform technology from Moderna. This work was done in parallel with the pre-clinical studies demonstrating the utility of delivering a stabilized coronavirus spike protein by mRNA delivery, providing the basis for the successful development of a SARS-CoV-2 mRNA-1273 vaccine (56, 59, 64–66). These studies provide a template and proof-of-concept for how combining technologies through public-private partnerships can provide solutions for future pandemic threats.

## Data Availability Statement

The raw data supporting the conclusions of this article will be made available by the authors, without undue reservation.

## Ethics Statement

The animal study was reviewed and approved by Animal Care and Use Committee of the Vaccine Research Center, NIAID, NIH.

## Author Contributions

RL, GS-J, JM, and BG designed initial protein constructs. SF, VP, EN, SH, and AC optimized mRNA design. RL, AD, TR, KM, YT, LK, JM, and BG designed research. RL, AD, TR, OA, LC, YT, RC, DN, GH, and GS-J performed research. RL, AD, TR, KM, YT, RC, LK, and BG analyzed and interpreted data. RL, AD, LK, and BG wrote the manuscript. RL, TR, KM, AD, LC, SH, AC, and BG helped edit the manuscript. All authors contributed to the article and approved the submitted version.

## Funding

This work was supported in part with federal funds from the Frederick National Laboratory for Cancer Research, NIH, under contract HHSN261200800001 (YT) and by the Intramural Research Program of the VRC.

## Conflict of Interest

Authors SF, VP, EN, SH and AC were employed by company Moderna Inc. YT is employed by Leidos Biomedical Research, Inc., supported in part with funds from the Frederick National Laboratory for Cancer Research, NIH, under contract HHSN261200800001. RL, GS-J, JM, and BG are inventors on patent applications involving Nipah virus vaccine designs.

The remaining authors declare that the research was conducted in the absence of any commercial or financial relationships that could be construed as a potential conflict of interest.

## Publisher’s Note

All claims expressed in this article are solely those of the authors and do not necessarily represent those of their affiliated organizations, or those of the publisher, the editors and the reviewers. Any product that may be evaluated in this article, or claim that may be made by its manufacturer, is not guaranteed or endorsed by the publisher.
