# Supplementary material for: Chimeric Fusion (F) and Attachment (G) Glycoprotein Antigen Delivery by mRNA as a Candidate Nipah Vaccine
Source: Front Immunol. 2021 Dec 8;12:772864. doi: 10.3389/fimmu.2021.772864 (PMC8692728; doi:10.3389/fimmu.2021.772864)
Supplement: Supplementary file 1 [file Presentation_1.pptx]

## Slide 1
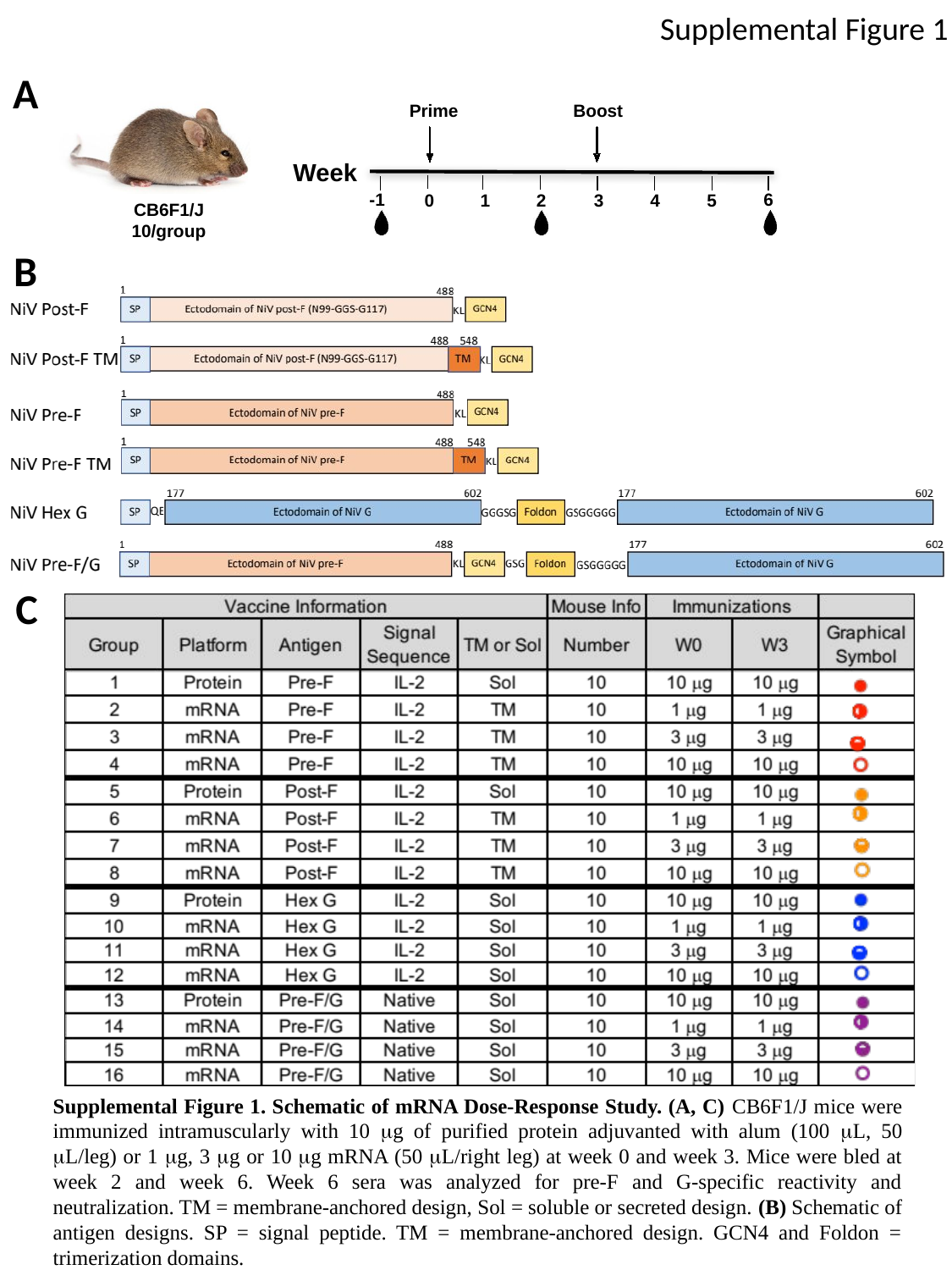

Supplemental Figure 1
A
Prime
Boost
Week
-1
6
0
1
2
3
4
5
CB6F1/J
10/group
B
C
Supplemental Figure 1. Schematic of mRNA Dose-Response Study. (A, C) CB6F1/J mice were immunized intramuscularly with 10 mg of purified protein adjuvanted with alum (100 mL, 50 mL/leg) or 1 mg, 3 mg or 10 mg mRNA (50 mL/right leg) at week 0 and week 3. Mice were bled at week 2 and week 6. Week 6 sera was analyzed for pre-F and G-specific reactivity and neutralization. TM = membrane-anchored design, Sol = soluble or secreted design. (B) Schematic of antigen designs. SP = signal peptide. TM = membrane-anchored design. GCN4 and Foldon = trimerization domains.

## Slide 2
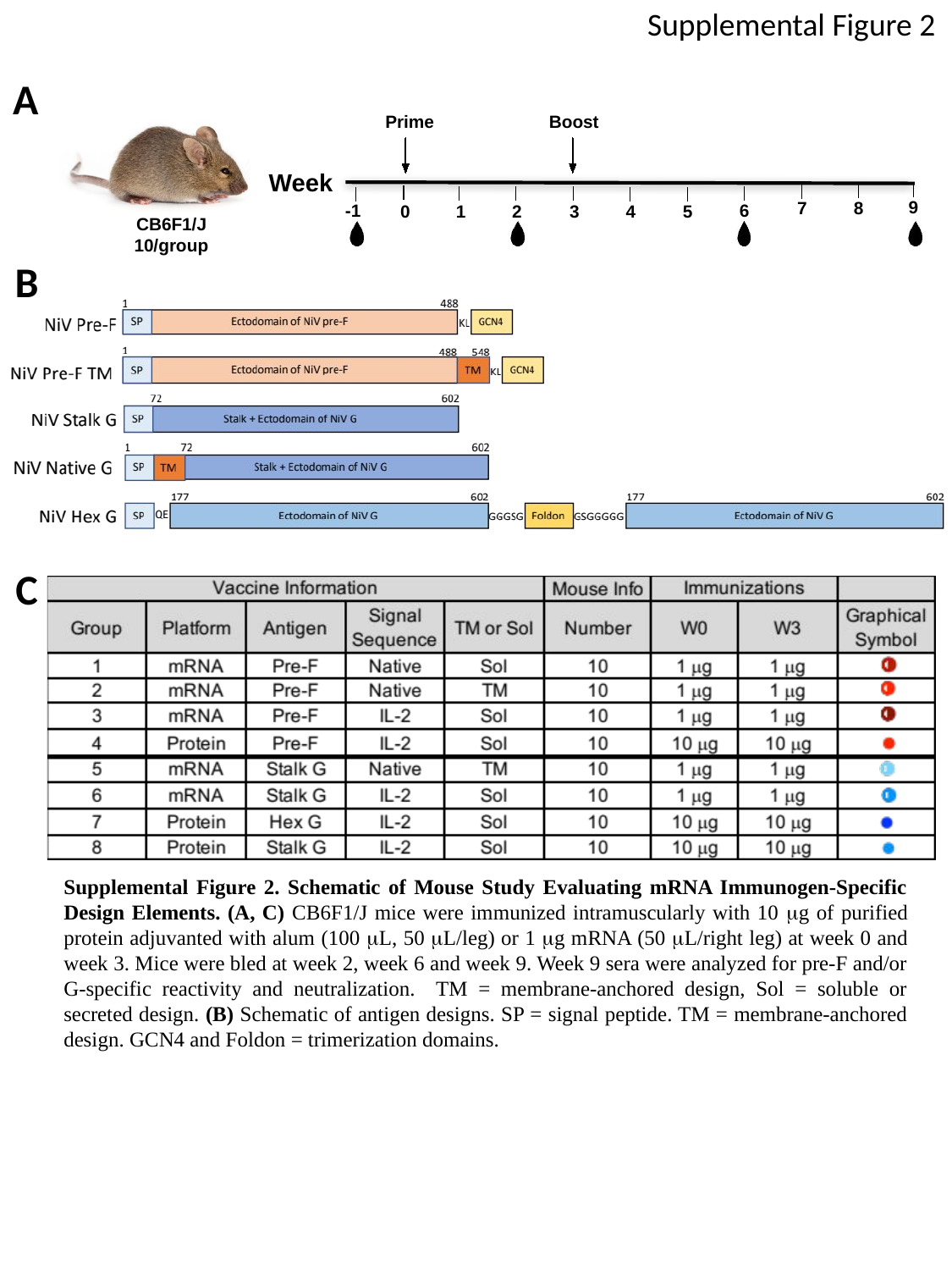

Supplemental Figure 2
A
Prime
Boost
Week
9
8
7
-1
6
0
1
2
3
4
5
CB6F1/J
10/group
B
C
Supplemental Figure 2. Schematic of Mouse Study Evaluating mRNA Immunogen-Specific Design Elements. (A, C) CB6F1/J mice were immunized intramuscularly with 10 mg of purified protein adjuvanted with alum (100 mL, 50 mL/leg) or 1 mg mRNA (50 mL/right leg) at week 0 and week 3. Mice were bled at week 2, week 6 and week 9. Week 9 sera were analyzed for pre-F and/or G-specific reactivity and neutralization. TM = membrane-anchored design, Sol = soluble or secreted design. (B) Schematic of antigen designs. SP = signal peptide. TM = membrane-anchored design. GCN4 and Foldon = trimerization domains.

## Slide 3
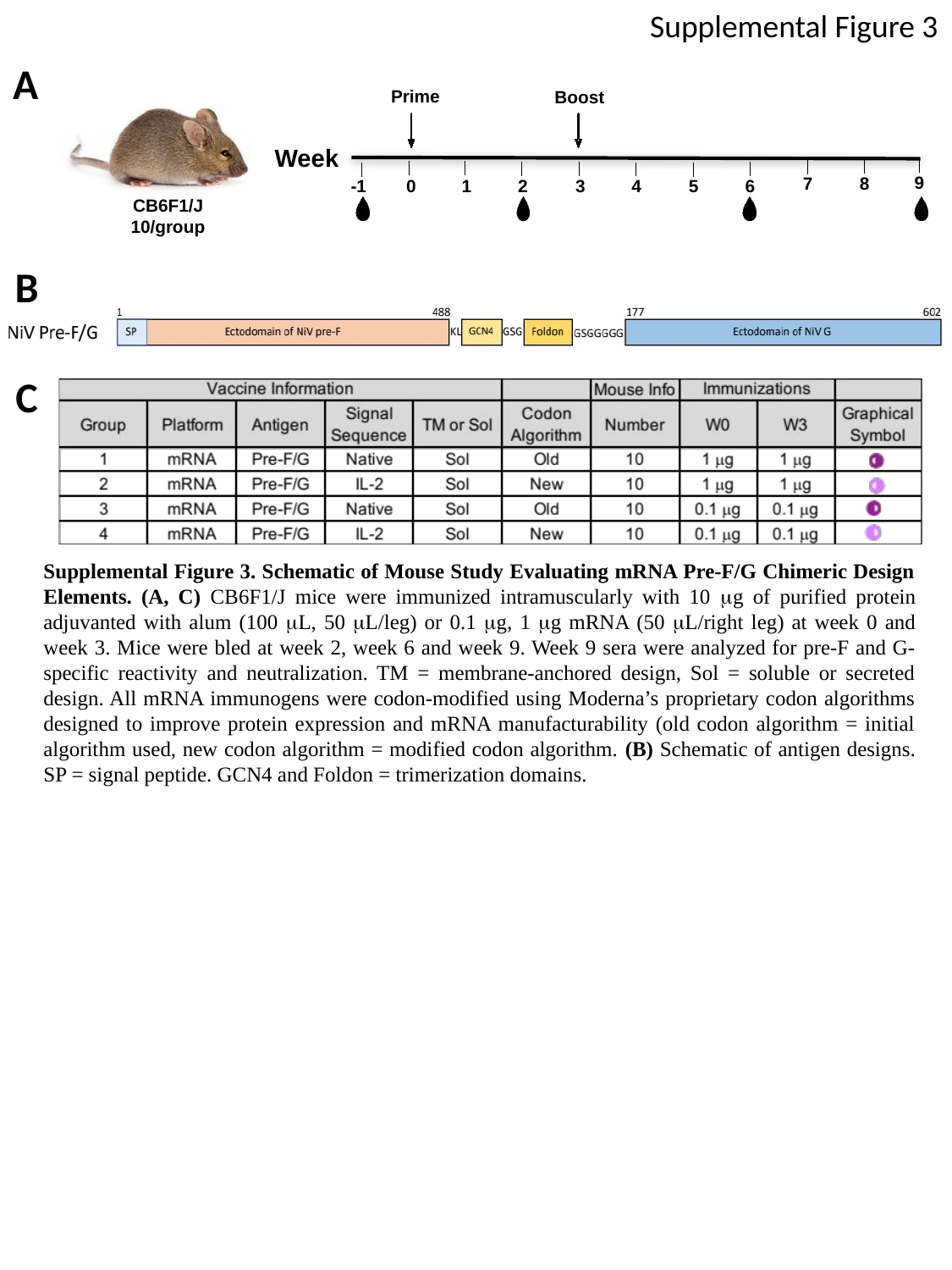

Supplemental Figure 3
A
Prime
Boost
Week
9
8
7
-1
6
0
1
2
3
4
5
CB6F1/J
10/group
B
C
Supplemental Figure 3. Schematic of Mouse Study Evaluating mRNA Pre-F/G Chimeric Design Elements. (A, C) CB6F1/J mice were immunized intramuscularly with 10 mg of purified protein adjuvanted with alum (100 mL, 50 mL/leg) or 0.1 mg, 1 mg mRNA (50 mL/right leg) at week 0 and week 3. Mice were bled at week 2, week 6 and week 9. Week 9 sera were analyzed for pre-F and G-specific reactivity and neutralization. TM = membrane-anchored design, Sol = soluble or secreted design. All mRNA immunogens were codon-modified using Moderna’s proprietary codon algorithms designed to improve protein expression and mRNA manufacturability (old codon algorithm = initial algorithm used, new codon algorithm = modified codon algorithm. (B) Schematic of antigen designs. SP = signal peptide. GCN4 and Foldon = trimerization domains.

## Slide 4
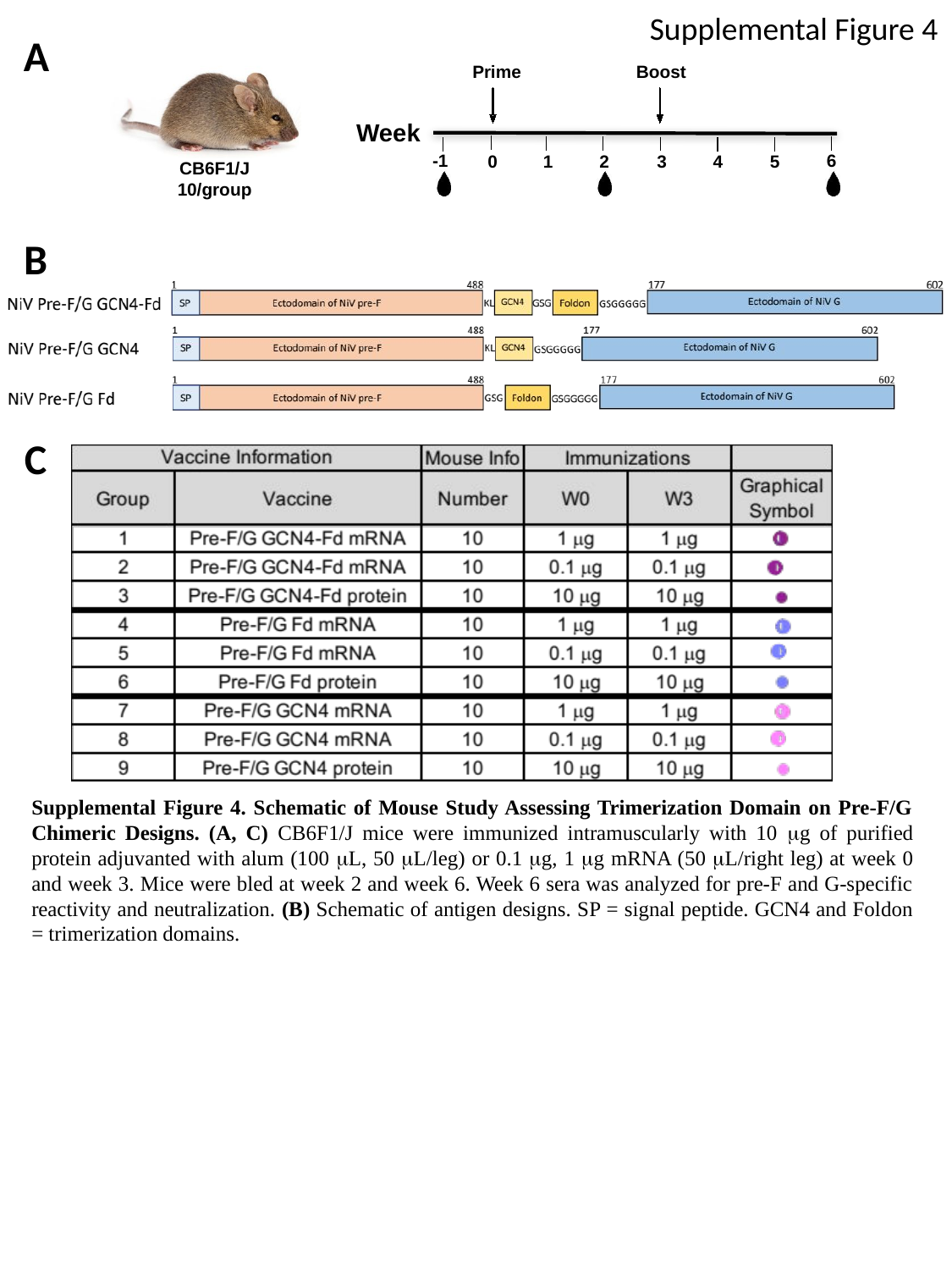

Supplemental Figure 4
A
Prime
Boost
Week
-1
6
0
1
2
3
4
5
CB6F1/J
10/group
B
C
Supplemental Figure 4. Schematic of Mouse Study Assessing Trimerization Domain on Pre-F/G Chimeric Designs. (A, C) CB6F1/J mice were immunized intramuscularly with 10 mg of purified protein adjuvanted with alum (100 mL, 50 mL/leg) or 0.1 mg, 1 mg mRNA (50 mL/right leg) at week 0 and week 3. Mice were bled at week 2 and week 6. Week 6 sera was analyzed for pre-F and G-specific reactivity and neutralization. (B) Schematic of antigen designs. SP = signal peptide. GCN4 and Foldon = trimerization domains.

## Slide 5
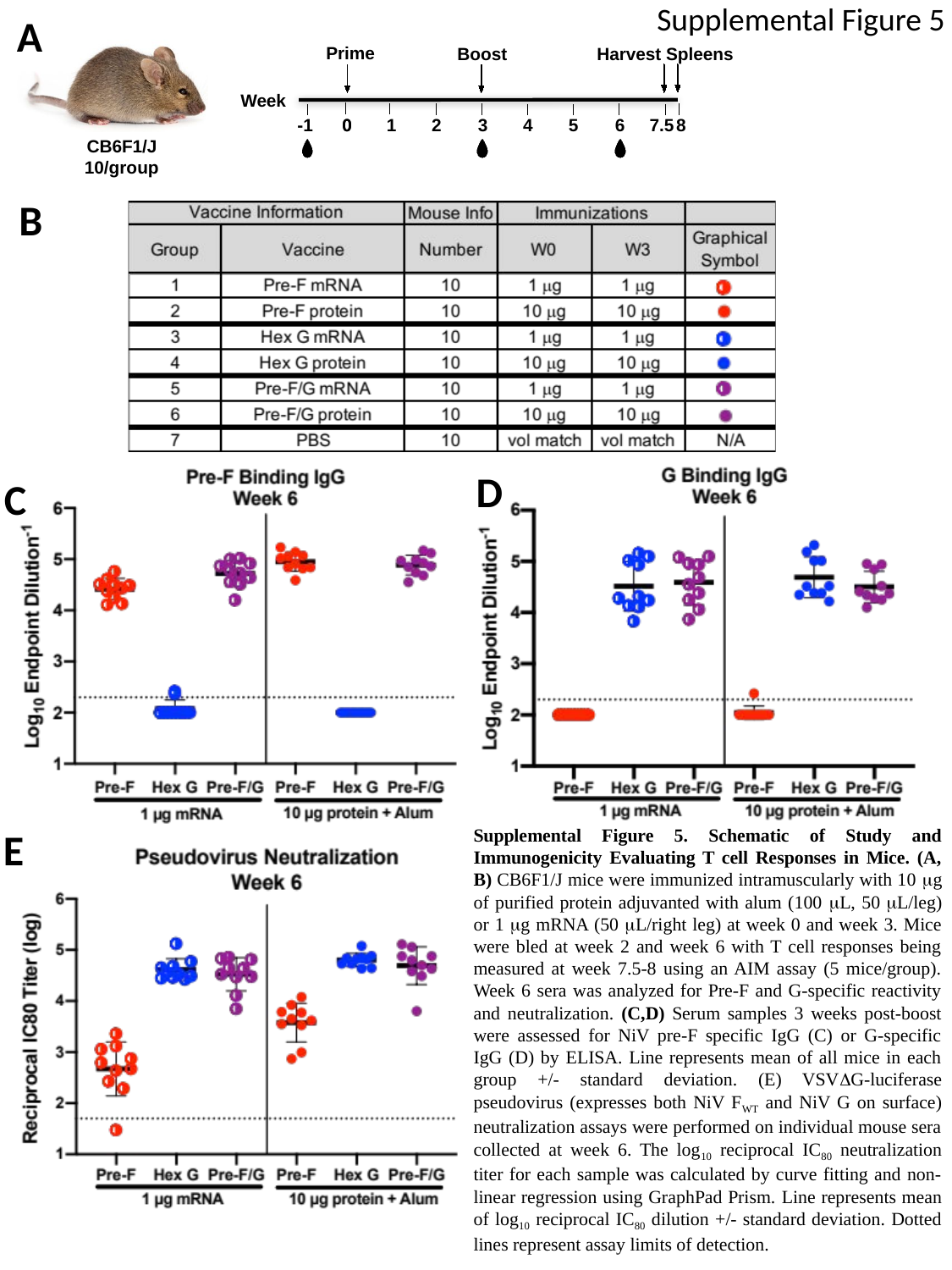

Supplemental Figure 5
A
Prime
Harvest Spleens
Boost
Week
-1
6
7.5
8
0
0
1
2
3
4
5
CB6F1/J
10/group
B
D
C
Supplemental Figure 5. Schematic of Study and Immunogenicity Evaluating T cell Responses in Mice. (A, B) CB6F1/J mice were immunized intramuscularly with 10 mg of purified protein adjuvanted with alum (100 mL, 50 mL/leg) or 1 mg mRNA (50 mL/right leg) at week 0 and week 3. Mice were bled at week 2 and week 6 with T cell responses being measured at week 7.5-8 using an AIM assay (5 mice/group). Week 6 sera was analyzed for Pre-F and G-specific reactivity and neutralization. (C,D) Serum samples 3 weeks post-boost were assessed for NiV pre-F specific IgG (C) or G-specific IgG (D) by ELISA. Line represents mean of all mice in each group +/- standard deviation. (E) VSVDG-luciferase pseudovirus (expresses both NiV FWT and NiV G on surface) neutralization assays were performed on individual mouse sera collected at week 6. The log10 reciprocal IC80 neutralization titer for each sample was calculated by curve fitting and non-linear regression using GraphPad Prism. Line represents mean of log10 reciprocal IC80 dilution +/- standard deviation. Dotted lines represent assay limits of detection.
E

## Slide 6
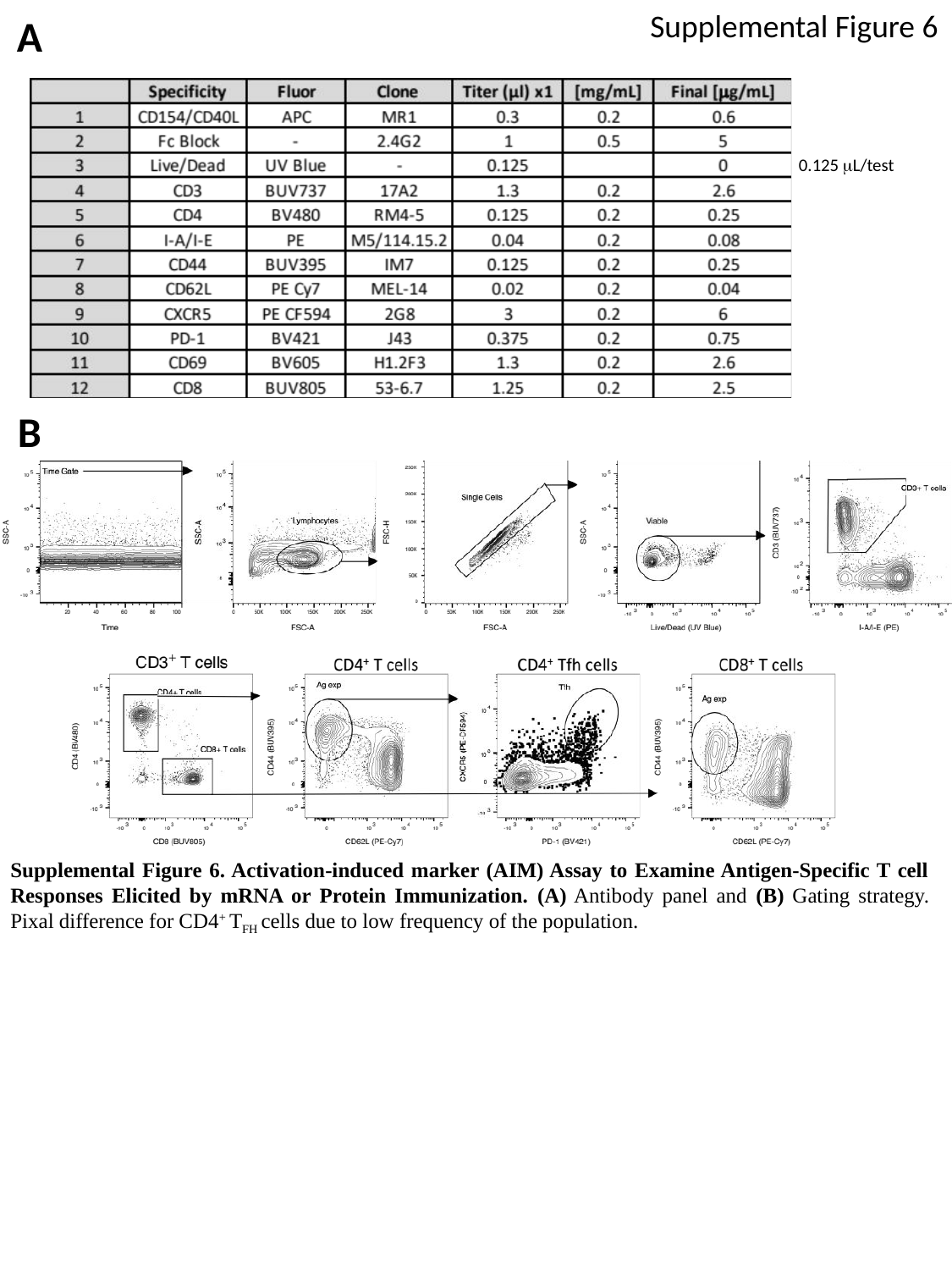

Supplemental Figure 6
A
0.125 mL/test
B
Supplemental Figure 6. Activation-induced marker (AIM) Assay to Examine Antigen-Specific T cell Responses Elicited by mRNA or Protein Immunization. (A) Antibody panel and (B) Gating strategy. Pixal difference for CD4+ TFH cells due to low frequency of the population.

## Slide 7
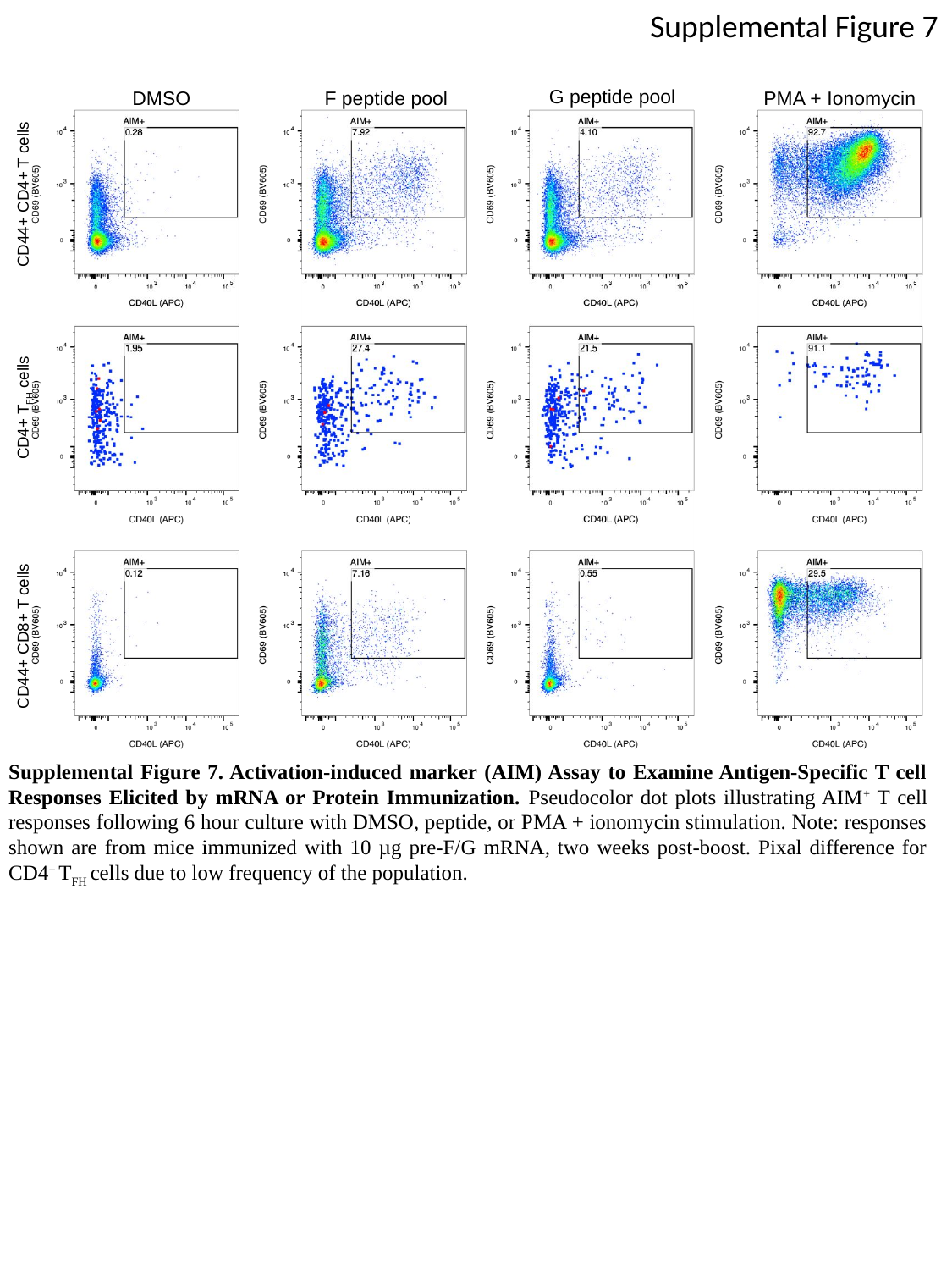

Supplemental Figure 7
G peptide pool
DMSO
F peptide pool
PMA + Ionomycin
CD44+ CD4+ T cells
CD4+ TFH cells
CD44+ CD8+ T cells
Supplemental Figure 7. Activation-induced marker (AIM) Assay to Examine Antigen-Specific T cell Responses Elicited by mRNA or Protein Immunization. Pseudocolor dot plots illustrating AIM+ T cell responses following 6 hour culture with DMSO, peptide, or PMA + ionomycin stimulation. Note: responses shown are from mice immunized with 10 µg pre-F/G mRNA, two weeks post-boost. Pixal difference for CD4+ TFH cells due to low frequency of the population.

## Slide 8
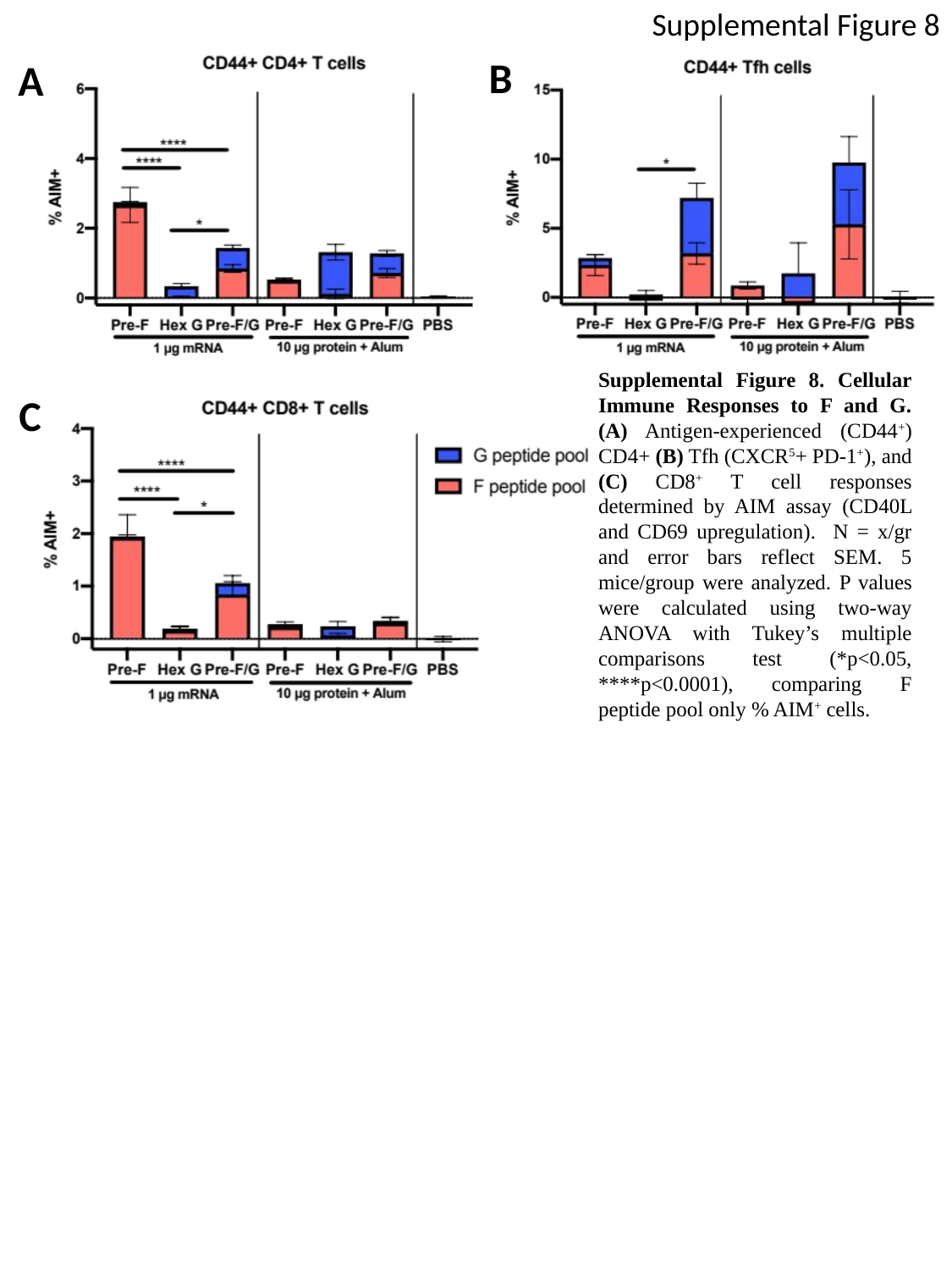

Supplemental Figure 8
B
A
Supplemental Figure 8. Cellular Immune Responses to F and G. (A) Antigen-experienced (CD44+) CD4+ (B) Tfh (CXCR5+ PD-1+), and (C) CD8+ T cell responses determined by AIM assay (CD40L and CD69 upregulation). N = x/gr and error bars reflect SEM. 5 mice/group were analyzed. P values were calculated using two-way ANOVA with Tukey’s multiple comparisons test (*p<0.05, ****p<0.0001), comparing F peptide pool only % AIM+ cells.
C

## Slide 9
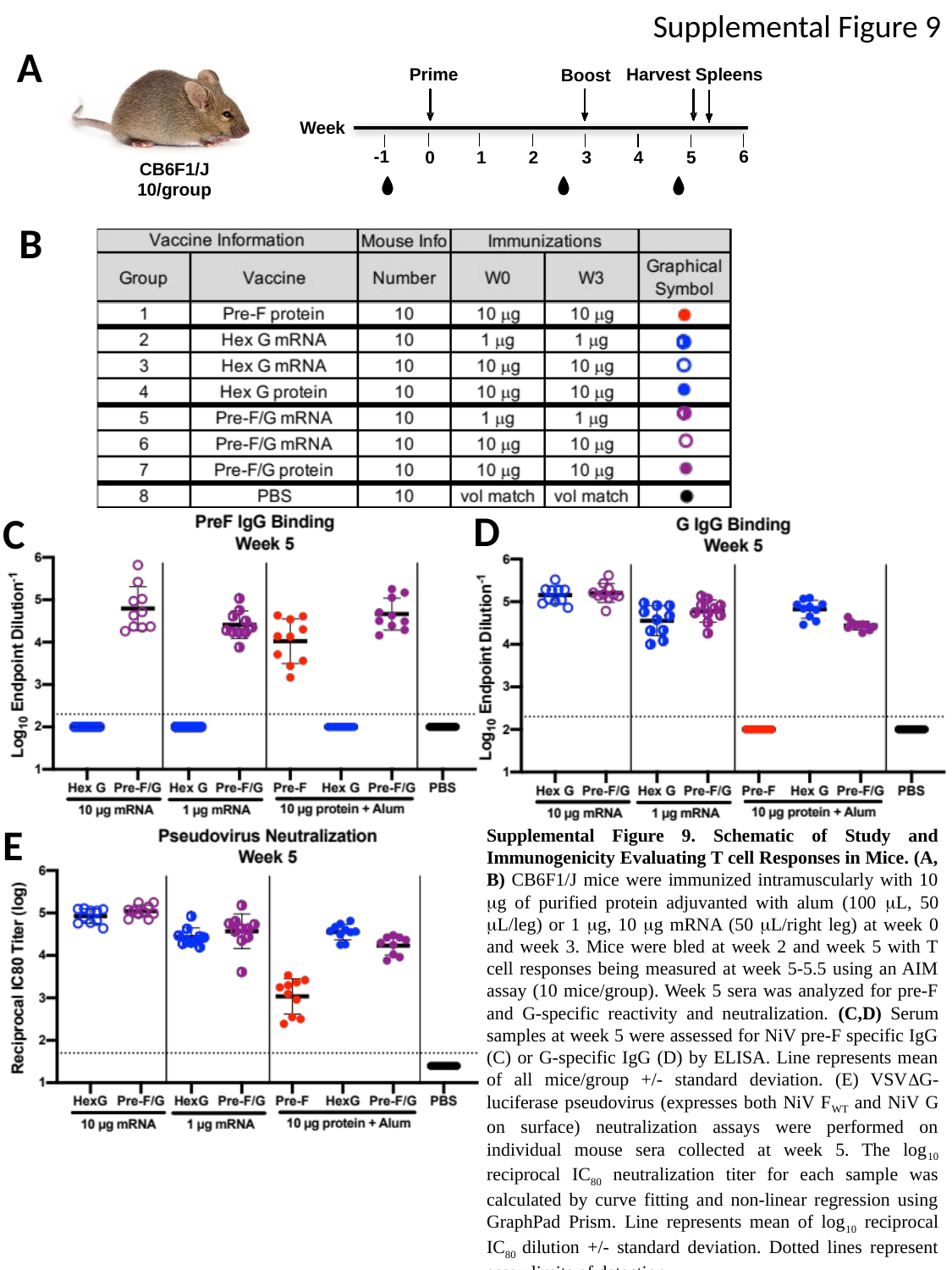

Supplemental Figure 9
A
Prime
Harvest Spleens
Boost
Week
-1
6
0
1
2
3
4
5
CB6F1/J
10/group
B
D
C
E
Supplemental Figure 9. Schematic of Study and Immunogenicity Evaluating T cell Responses in Mice. (A, B) CB6F1/J mice were immunized intramuscularly with 10 mg of purified protein adjuvanted with alum (100 mL, 50 mL/leg) or 1 mg, 10 mg mRNA (50 mL/right leg) at week 0 and week 3. Mice were bled at week 2 and week 5 with T cell responses being measured at week 5-5.5 using an AIM assay (10 mice/group). Week 5 sera was analyzed for pre-F and G-specific reactivity and neutralization. (C,D) Serum samples at week 5 were assessed for NiV pre-F specific IgG (C) or G-specific IgG (D) by ELISA. Line represents mean of all mice/group +/- standard deviation. (E) VSVDG-luciferase pseudovirus (expresses both NiV FWT and NiV G on surface) neutralization assays were performed on individual mouse sera collected at week 5. The log10 reciprocal IC80 neutralization titer for each sample was calculated by curve fitting and non-linear regression using GraphPad Prism. Line represents mean of log10 reciprocal IC80 dilution +/- standard deviation. Dotted lines represent assay limits of detection.
